# Child Immunization Coverage in Urban Settings of Twelve Provinces Plus Kabul, Afghanistan, 2019

**DOI:** 10.1155/2024/5400013

**Published:** 2024-08-14

**Authors:** Khwaja Mir Islam Saeed, Shoaib Naeemi, Ruqia Naser, Bahara Rasooly, Mir Salamuddin Hakim, Khalid Arman, Homeira Nishat

**Affiliations:** ^1^ Afghanistan Field Epidemiology Training Program (AFETP) Eastern Mediterranean Public Health Network (EMPHNET), Kabul, Afghanistan; ^2^ Feild Epidemiology Afghanistan National Public Health Institute (ANPHI), Kabul, Afghanistan; ^3^ Immunization Expert, Kabul, Afghanistan; ^4^ UNICEF Polio Section World Health Organization, Kabul, Afghanistan

**Keywords:** Afghanistan, coverage, factors, immunization, urban

## Abstract

**Background:** Low immunization and discrepancies in data sources have been a consistent challenge in Afghanistan. The objective of this was to estimate the coverage of immunization status among children of 12–23 months in urban settings of 12 provinces plus Kabul, Afghanistan in 2019.

**Methods:** A cross-sectional survey was conducted in the capital of 12 cities of polio high-risk provinces plus Kabul during October–December 2019. A two-stage cluster sampling was used to approach 30 clusters and interview seven households. The coverage for 13 vaccines against 10 childhood diseases prioritized by the Afghanistan Immunization program was assessed through observation of vaccine cards or by history from caregivers of children. Epi Info v.7.2.5 was used for data management and analysis.

**Results:** Totally, 3382 caregivers of children aged 12–23 months, of whom 50.8% were boys, were interviewed. The literacy of mothers was 35%, and 86.4% were housewives with no formal employment. The average age of children was 17.07 ± 4.05 months. In total, 1261 (37.29%) children were fully vaccinated, 833 (54.2%) were partially vaccinated, and 288 (8.52%) did not receive any dose of routine vaccine. Of total, 71.82% had vaccination cards, 17.24% had lost them, and 11% had no cards. Generally, coverage of immunization by cards and history was 91.70% for BCG, 52% for Penta, 78% for OPV-4, 63% for PCV2, 61% for Rota2, 68.50% for measles 1, and 58% for IPV. Nangarhar and Kunar provinces have the highest and lowest immunization coverage, respectively. Lack of awareness and time was the main factor cited by partially vaccinated individuals, while misconceptions about vaccines were reported among the unvaccinated.

**Conclusion:** Child immunization levels, varying across cities, were suboptimal in the study population. Realistic goal-setting and awareness campaigns are necessary to address the low immunization coverage and fight against barriers in Afghanistan.

## 1. Introduction

Immunization is one of the most cost-effective public health interventions to date, averting millions of deaths annually, and has led to the control and eradication of certain infectious diseases [1]. According to modeling the impact of the global immunization agenda for 2030, vaccines could avert at least 5–5.4 million deaths per year due to vaccine-preventable diseases between 2021 and 2023 [2]. Global coverage rates for the third dose of the diphtheria, tetanus, and pertussis vaccine (DPT3) as an indicator of immunization coverage reached 85% in 2018, up from 72% in 2000 and compared to 21% in 1980 [3]. However, with the emergence of the COVID-19 pandemic, disruptions were made in the path of routine immunization coverage decreasing the rate of DPT3 to 83% in 2020 and 81% in 2021 [4]. The decline in routine immunization coverage was not limited to DPT3 during the pandemic since other routine vaccines witnessed a decline in the global level. For instance, the global coverage for measles-containing vaccine (MCV1) was reportedly 81% showing a 4% decline compared to 2019, while coverage for oral polio vaccine (OPV) reached 80% in 2021 compared to 85% in 2018. Furthermore, in 2021, around 18 million children were classified in the zero-dose category with 5 million (27%) children coming from low-income countries [5].

Despite the efforts made postpandemic to leverage the immunization coverage globally to that of prepandemic, still, the recovery is suboptimal and uneven based on regions and countries. Immunization efforts and priorities were somehow shifted to COVID-19 vaccination worldwide and rooted for relatively slower recovery. For instance, DPT3 coverage slightly increased to 84% in 2022 compared to 81% in 2021. Furthermore, the number of zero-dose children decreased from 18.1 million in 2021 to 14.3 million in 2022, yet not reaching the prepandemic level of 12.9 million in 2019. The first dose of the measles vaccine increased from 81% in 2021 to 83% in 2022; however, a staggering 21.9 million children missed their first dose of the measles vaccine [6]. Moreover, in 2022, 20.5 million children were accounted as un/undervaccinated globally [7].

Considering the context of the Immunization Agenda 2030 held in 2021, 20 countries across the globe were prioritized to enhance their immunization coverage. Afghanistan, being part of the Eastern Mediterranean Region (EMR) of World Health Organization (WHO), is among 20 countries with immunization priority with a ranking of 13 in 2021 and 12 in 2022 [6]. Despite inconsistency in sources, still among neighboring countries, Afghanistan has the lowest routine coverage, according to WHO-UNICEF reports: the coverage rates of DPT3 in Iran, Pakistan, and Afghanistan are estimated at 99%, 85%, and 69%, respectively, as of 2023 [8].

In Afghanistan, the immunization program started in the year 1978 in the name of “Mass Immunization Program” and has been functioning in the structure of the Ministry of Public Health (MoPH) striving to achieve universal immunization throughout the country as the National Expanded Program on Immunization (NEPI). In 2003, the immunization program was included in the MoPH structure. Preliminary, expanded program on immunization (EPI) services were comprised of providing six routine vaccines, to which later hepatitis B, *Haemophilus* influenzae type B (Hib), pneumonia conjugated vaccine (PCV13), Hep-B (birth dose), injectable polio vaccine (IPV), and Rota vaccines were introduced to the routine immunization schedule in 2006, 2009, 2013, 2014, 2015, and 2018, respectively [9]. Currently, NEPI routine programs throughout Afghanistan apply 13 vaccines against 10 childhood diseases (Table 1). Five of these vaccines (diphtheria, pertussis, tetanus, Hep-B, and Hib) are prepared together and applied as Penta (replaced with DPT). Moreover, measles, Hep-B (birth dose), IPV, OPV, BCG, PCV13, Rota, and tetanus toxoid (TT) for childbearing age (CBA) women are also provided by MoPH. Children aged 0–11 months are targeted for routine EPI.

Afghanistan consists of 34 provinces, around 400 districts, and close to 3000 EPI service points. Three main strategies are implemented to provide immunization services to children throughout the country under the names of fixed (clients come to a health facility with 0.5–1-h walking distance), outreach (vaccinator goes to the field/village and returns on the same day), and mobile (vaccinator goes to the field and needs an overnight stay indicating long distance). Along with fixed strategy, the EPI services are provided by outreach and mobile strategies. The outreach and mobile strategies are mostly implemented in the rural areas, while in the urban areas and big cities, most of the targeted population is covered by the fixed strategy [10]. The progressive high-density suburbs of the big cities and establishment of new settlements in the cities remain a challenge to reach all targeted children and women. As urbanization accelerates, the population of persons in slum environments also keeps increasing especially in rapidly growing medium and large urban areas in Asia particularly in Afghanistan. Thus, a large proportion of children and women targeted for vaccinations will be residing in urban areas with a significant proportion in disadvantaged, poor, and underserved communities [11].

The Afghanistan Household Survey (AHS 2018), which was comparing the national immunization coverage between urban and rural areas, reflected that the rural areas, generally, are slightly behind urban areas (68.6% vs 58.4%) [12]. However, the Multiple Indicator Cluster Survey (MICS) held by UNICEF for the period of 2022–2023 revealed that only 36.6% of children aged 12–23 months received basic immunization (including BCG, OPV, MCV1, and DPT3) while the proportion of children aged 24–35 months receiving full vaccination was 16.2%. In resemblance with the AHS 2018 report, findings of MICS indicated slightly higher basic immunization coverage in urban areas (49.6%) compared to rural (32.5%) [13]. In addition to that, the result of knowledge, attitude, and practice (KAP) survey-2018 showed that 72% of female caregivers thought that vaccinations were suitable for prevention of diseases out of whom 43.5% correctly highlighted that children needed to have five vaccinations before 12 months. Seventy-one percent reported that they had fully vaccinated their child. The full vaccination rates were higher among urban respondents than the rural respondents (85.2% and 78.4%, respectively) [14].

The data obtained from recent surveys, while valuable in terms of quantifying immunization coverage rates, lacks the ability to attain specified coverage targets. Furthermore, these surveys were of a multi-indicator nature, employing extensive questionnaires that offer a broad overview of health-related information rather than specific, program-focused details, including the intricacies of the EPI. The objective of this was to estimate the coverage of immunization status among children of 12–23 months in 12 urban settings of Afghanistan in 2019.

## 2. Materials and Methods

After planning and approval of research protocol, we conducted a descriptive cross-sectional study in urban areas of 12 high-risk provinces for polio plus Kabul in Afghanistan. The provinces were selected based on the abundance of polio virus-reported cases across the country during the last decade while the risk for polio virus transmission has been triggered due to the neighboring geography of these provinces with Pakistan, another polio endemic country. The main target populations were children 12–23 months living in urban areas to represent the births in a 1-year period—an annual birth cohort. The eligibility criteria for enrollment of mentioned children living in urban settings for more than a year agree to participate in the study. However, we excluded those in doubt of age range (not possessing vaccine card, birth certificate, or accurate guess of caretaker about the age of the child), guests, the families who did not agree to participate, and areas with insecurity and harsh geographical areas (mobile villages and areas without transportation access). The study was conducted to interview the mother or caretaker of children, so the female presence was very much needed.

**Figure 1:**
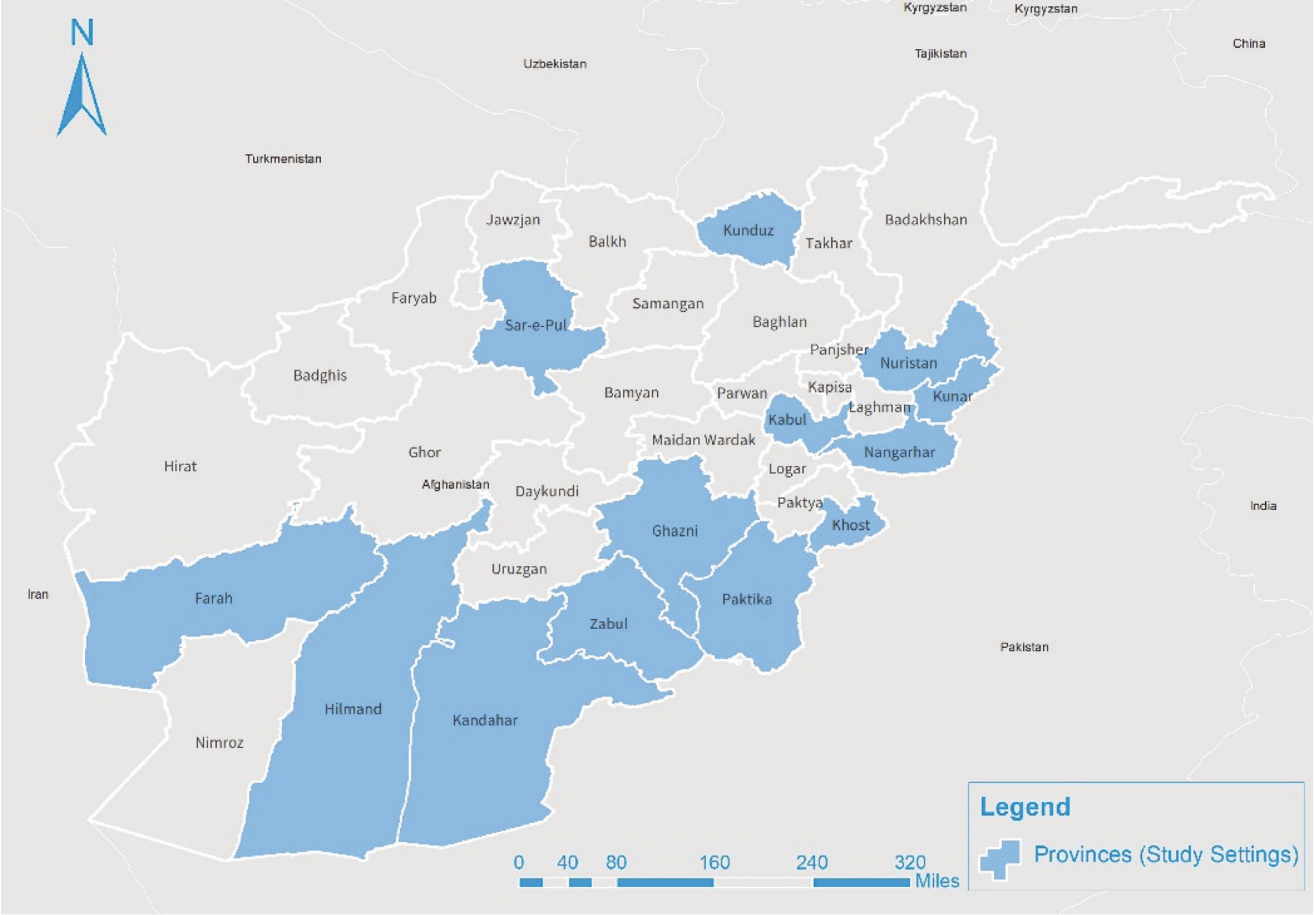
Location of 12 provinces and capital surveyed for immunization coverage in the map of Afghanistan.

The survey was conducted by residents of the Afghanistan Field Epidemiology Training Program (AFETP) in urban areas of 12 high-risk provinces plus Kabul. Here are the sample size and provinces from which the residents came to the training program such as Kandahar (447), Zabul (218), Helmand (241), Farah (170), Sarepul (210), Kunduz (207), Nangarhar (203), Nuristan (95), Kunar (220), Paktika (224), Khost (216), Ghazni (217), and Kabul (714) (Figure 1).

As recommended by the WHO, we conducted a two-stage cluster sampling strategy using the WHO EPI 30 cluster and 7 households (210) 2-step method, described in the WHO document “WHO/IVB/04.23 Immunization coverage survey” [15]. Apparently, the total sample size was calculated as 2520 households of children aged 12–23 months (7 × 30 × 12). The sample size, for Kabul, was tripled and doubled for Kandahar to compare to other provinces due to its more condensed urban population; so, the final sample size as of calculation was 3150; however, the size was exceeded to 3382 households on the ground. Initially, we selected 30 urban clusters randomly from the available EPI list in each province, and later, the team approached the center of each area or cluster and randomly chose to go north, south, east, or west for the selection of households. In the second phase, seven households per cluster were selected randomly per cluster and asked for an interview with the mother or attendant of children between 12 and 23 months. If the first house has no eligible child, then the surveyor moved to the closest house until they found the right respondents.

Data were collected by trained interviewers through a face-to-face interview using a structured questionnaire (appendix 1) of local languages in which the informed consent was also accommodated. The questionnaire contained demographic including age, sex, education, occupation, and general information; questions on coverage of vaccination by both card and history; and collected data on factors associated with immunization using open-ended questions. The team asked for vaccination cards of the children aged 12–23 months and checked the immunization status for each of the provided antigens, namely, BCG, Pentavalent, OPV, Rota, PCV, and the first dose of measles vaccines. AFETP technical team, provincial office, and residents themselves either collected data or supervised the process to ensure quality when female data collectors were hired. Furthermore, data collectors checked the questionnaire forms once they were completed for any discrepancy or unfilled fields to rectify them. So, they were responsible for the accuracy of data before data entry.

For the sake of this study, the following operational definitions were used as defined by the NEPI of Afghanistan:

1. Fully immunized: A child can be defined as fully immunized if they have received a BCG, three doses of the Penta vaccine (diphtheria, pertussis, tetanus, Hep-B, and Hib), three doses of OPV, and a measles vaccine and should be fully immunized within the 1-year child of life (11 months of age).
2. Partial immunized: Partial immunization refers to a child who has not received one or more of the recommended above-mentioned doses of a vaccine according to the prescribed vaccination schedule.
3. Unvaccinated: Unvaccinated or no immunization means child who has not received even a single dose of vaccine.
4. Dropout: Immunization dropout signifies that the child has received the first recommended dose of the vaccine yet has missed the next recommended dose.

While the data collection was over, all questionnaires were brought to the AFETP residents' office in each province, where cleaning took place. Data management and analysis were done using Microsoft Excel 2016 and Epi Info version 7.2.5. Frequency distributions of all the independent variables were generated to present the sociodemographic characteristics of the respondents. Summary statistics including mean, standard deviation, and range was calculated for the continuous variables, for example, age. The prevalence of outcome variables (vaccination coverage) was calculated as a percentage. The protocol was submitted to the institutional review board (IRB) of the Afghanistan National Public Health Institute (ANPHI), and approval was taken.

## 3. Results

In this study, the data from urban settings of 12 provinces plus Kabul were collected and analyzed. In total, 3382 households were visited, and one eligible child of 12–23 months from each household was interviewed. From the total number of caretakers who participated in this study, 2622 (78%) were mothers, 289 (8.5%) were fathers, and the rest were other relatives of the eligible children. The mean age of children was 17.07 ± 4.05 months of whom 1713 (50.8%) were males. Among caretakers, 2188 (64.95%) were illiterate compared to 1181 (35.05%) of literate. The basic characteristics of the study population are presented in Table 2.

In total, 1261 (37.29%) children were fully vaccinated, 1833 (54.2%) were partially vaccinated, and 288 (8.52%) were unvaccinated receiving no vaccine at all. Out of all respondents, 2421 (71.82%) had vaccination cards, 581 (17.24%) expressed that they had cards but could not show them, and 369 (10.95%) did not have any vaccination cards. The study found that, aggregately based on card and history, the BCG vaccine had a coverage of 2400 (71%), while Penta-1 and Penta-3 had coverages of 2300 (68%) and 1998 (59%), respectively (Table 3). The results indicated the low coverage rates of OPV, specifically OPV-0, OPV-1, OPV-2, OPV-3, and OPV-4. For OPV-0, the coverage rate was recorded as 2274, which corresponds to 67% of the target population. Similarly, for OPV-1, the coverage rate was slightly higher at 2322, representing 68% coverage. However, the coverage rate for OPV-4 was the highest among these vaccines, with 2461 individuals vaccinated, reaching a coverage rate of 73%. Interestingly, when comparing the coverage rates for polio vaccine, OPV-4 has the highest coverage rate based on history (732, 21.6%) compared to others. The results also revealed that among the target population, the coverage rate of measles 1 vaccine by card was 1795, indicating a coverage rate of 53%. An additional analysis of the coverage rate based on history demonstrated that the coverage rate of measles 1 vaccine was 520, accounting for 15% of the target population in the 12 provinces. The results indicated that a significant proportion of the target population remains unvaccinated regarding IPV, PCV3, and Penta-3. For IPV, the coverage rate shows that 1413 individuals, accounting for 42% of the target population, have not received the vaccine. Similarly, the data for PCV3 revealed that 1389 individuals, representing 41% of the target population, have not been vaccinated with this vaccine. Furthermore, the coverage rate for Penta-3 indicated that 1384 individuals, approximately 41% of the target population, have not received the required three doses of the vaccine. Table 3 delves deeper in the coverage of routine vaccines differentiated by card check, history, and not vaccinated.

At the provincial level, the number of households differed due to the proportion of population in provinces. Kabul, as the capital, had the highest, and Kandahar came second, while others had the same sample sizes, except Nuristan with the lowest proportion of households. Immunization coverage however differed based on provincial level. Interestingly, the province of Nangarhar located in the eastern part of Afghanistan reported the highest levels of coverage for multiple vaccines, including 98% for BCG, 89.7% coverage for the Rota2 vaccine, 89.3% coverage for the OPV3, 87.7% coverage for Penta-3, and 78.3% coverage for measles 1. In contrast, the coverage rates for essential childhood vaccines remained alarmingly low in Kunar Province. The BCG vaccine had a coverage rate of only 41%, while the coverage rates for Rota2, OPV3, and Penta-3 vaccines were even lower at 40% and 39.5%, respectively. Yet, the coverage rate for the measles 1 vaccine was comparatively higher at 80%. However, for the capital, Kabul, the measles 1 vaccine had a coverage of 78% followed by BCG at 70% and Penta-3 at 65%. The coverage rate for BCG was determined to be 71% in Kandahar with Penta-3 and measles 1 having a coverage rate of 58% and 65%, respectively (appendix 2).

**Figure 2:**
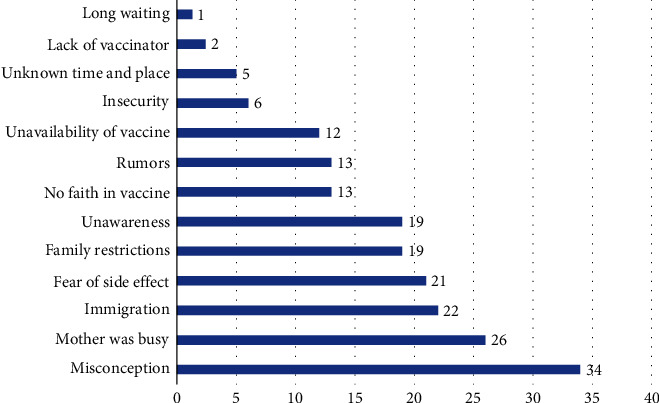
Factors leading to partial vaccination in urban settings, Afghanistan.

In addition to assessing the rates of immunization coverage, this study delved into the factors influencing parents' decisions regarding full, partial, or nonvaccination of their children. Among those who reported fully vaccinating their children, the most cited reason was a recognition of the crucial importance of vaccination, with 690 (54.71%) of respondents acknowledging this factor. Other motivating factors included the convenience of proximity to a healthcare facility offering vaccines (72, 7.78%) and the effective outreach efforts of vaccination programs (23, 1.82%). Furthermore, a positive interaction with vaccinators was mentioned by some parents as a contributing factor to their decision to vaccinate their children (22, 1.74%). These factors stem from the analysis of responses provided by a total of 1261 individuals who reported full immunization. On the other hand, the study revealed that parents or caretakers of partially vaccinated children reported the presence of various factors contributing to incomplete vaccination. Among this group, approximately 254 (14%) of individuals cited unawareness as a significant reason for partial vaccination. Additionally, 239 (13%) of caregivers expressed a lack of time as a substantial barrier in completing their children's vaccination schedule. Furthermore, a notable 175 (10%) of respondents mentioned losing their child's vaccination card as a factor contributing to incomplete vaccination. Some other factors such as insecurity of accessing health facilities for vaccination and wide time gaps between vaccinations were reported as well. Furthermore, the analysis identified several other factors including not having family permission and closure of health facility among 59 (3%) of partially vaccinated children (Figure 2).

The data reveals that individuals who chose not to take the vaccine provided various reasons for their decision. From the total of 288 unvaccinated children's primary caretakers, the most prevalent factor, accounting for 99 (34%), was the presence of misconceptions and incorrect ideas about the vaccine. Such misconceptions were mainly fear of causing infertility among children after taking the vaccine. Additionally, respondents also believed that the vaccine is “Haram” or prohibited in Islam which could be translated as misconception.

**Figure 3:**
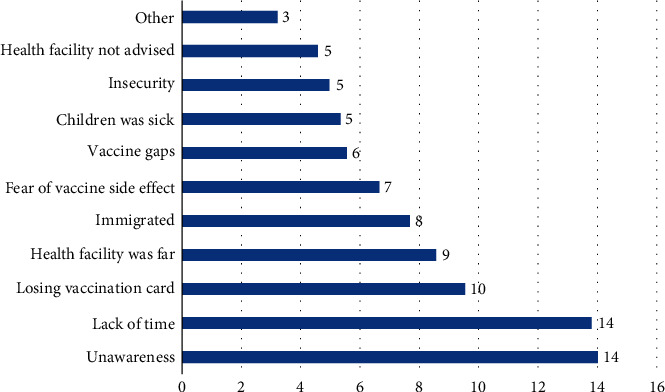
Factors reported for no vaccination in urban settings, Afghanistan.

Following closely behind was the occupational engagement of mothers, with 75 (26%) of respondents citing this as a contributing factor. Immigration also accounted for 64 (22%) of the caretakers/parent's reasons for not taking the vaccine. Additionally, 63 (22%) expressed fear of vaccine side effects, while 40 (13%) mentioned hearing false rumors as influencing their decision. Respondents reported hearing the rumor that vaccines are overseas' products and being administered to Afghans as part of their conspiracy towards Afghanistan which eventually affected and influenced their decision. Caretakers also reported familial restrictions as the factor for not vaccinating the children. This restriction has been defined as father, grandfather, and close family relatives not giving the permission to vaccinate the children. Interestingly, only a small percentage, 7 (2.4%), cited the lack of availability of vaccinators as a factor for not taking the vaccine. Other factors such as insecurity of accessing health facilities for vaccination were reported as well (Figure 3).

## 4. Discussion

Vaccination coverage is a critical indicator of the success of immunization programs in protecting populations against vaccine-preventable diseases. In this study, we assessed the vaccination coverage among children aged 12–23 months in urban settings of 12 provinces plus Kabul, Afghanistan. Our findings shed light on the status of immunization coverage and highlight important factors influencing parents' decisions regarding vaccination.

It is assumed that due to various reasons, the coverage of immunization would be higher in urban settings as compared to rural areas. Such differences have been reported in various settings including Afghanistan [8, 16, 17]. The overall vaccination coverage in the study population was suboptimal, with only 37.29% of children being fully vaccinated which is very far from the target of 80%. These findings were lower as compared to other countries including India [18] and Ethiopia [19]. Additionally, 54.2% of children were partially vaccinated, indicating that a significant proportion of the target population remained unprotected against vaccine-preventable diseases. These results underscored the need for focused efforts to improve immunization coverage and ensure that all children receive the recommended vaccines.

The coverage rates varied across different vaccines. BCG, a vaccine against tuberculosis, had the highest coverage rate at 71%. This finding suggests that efforts to administer BCG have been relatively successful, potentially due to its inclusion in the routine immunization immediately after birth to all children and the recognition of tuberculosis as a significant health concern in Afghanistan. However, it is crucial to note that even with the relatively high coverage rate, almost 30% of children did not receive this vaccine.

The coverage rates for other vaccines were notably lower. Penta-1 and Penta-3, vaccines that protect against diphtheria, tetanus, pertussis, hepatitis B, and Hib, had coverage rates of 68% and 59%, respectively. These rates indicated a substantial number of children that are vulnerable to these infectious diseases.

Similarly, the coverage rates for OPV varied across different doses, ranging from 67% for OPV-0 to 73% for OPV-4. Although OPV-4 had the highest coverage rate, it is concerning that a significant proportion of children did not receive the earlier doses, leaving them susceptible to polio. This coverage rate regarding polio vaccines is worrying because Afghanistan remains as one of the endemic countries for polio virus. A vast effort to eradicate polio has been made in Afghanistan through implementing vaccination campaigns across the country. Such efforts may lead to false assumption of the vaccinators to neglect polio vaccine administration leading to its lower vaccination coverage. However, the assumed reason for such low vaccination coverage rate of polio vaccine requires further studies, particularly focusing on this issue. Measles 1 vaccine had a coverage rate of 53% based on vaccination cards and 15% based on historical data, indicating a considerable gap in protection against measles.

The provincial-level analysis revealed disparities in vaccination coverage between provinces. Nangarhar province reported the highest coverage rates for multiple vaccines, including BCG, Rota2, OPV3, Penta-3, and Measles 1, suggesting relatively successful immunization efforts in that region. On the other hand, Kunar province had alarmingly low coverage rates for essential childhood vaccines, such as BCG, Rota2, OPV3, and Penta-3. Interestingly, these two provinces have a variety of similarities including geographical border, located across the border of Afghanistan-Pakistan as well as cultural context. However, the implementing organizations (nongovernmental organizations) for EPI programs differ for both provinces which may act as a reason for such discrepancies. These disparities highlight the need for targeted interventions and further studies to be conducted to address the barriers and challenges specific to each province particularly those with lower vaccination rate and ensure equitable access to immunization services.

Understanding the factors influencing parents' decisions regarding vaccination is crucial for developing effective strategies to improve coverage. Among those who reported full vaccination of their children, the recognition of the importance of vaccination was the most cited motivating factor. The convenience of proximity to healthcare facilities and effective outreach efforts of vaccination programs also played a role in parents' decision-making. Positive interactions with vaccinators were mentioned as contributing factors as well. Conversely, parents of partially vaccinated children cited factors such as unawareness, lack of time, and losing vaccination cards as barriers to completing their children's vaccination schedule. Other hindering factors included insecurities, having a sick child, and health facility closures. These findings emphasize the importance of targeted education and awareness campaigns, improving access to vaccination services, and implementing systems to mitigate the loss of vaccination cards. Individuals who chose not to vaccinate their children provided various reasons for their decision. Misconceptions and incorrect ideas about vaccines were the most prevalent factor, highlighting the need for effective communication. However, low distribution of partially and unvaccinated children among study population limited the implementation of bivariate and multivariate analysis regarding inference on hindering factors. Further studies are required particularly focusing on this issue in the context of Afghanistan.

Despite providing important results, this study was limited in providing results from the national level as well as rural areas. In addition, low resources and time limitation restricted the FETP residents to collect data from rural settings. In addition, the study did not include the immunization coverage of mothers including TT vaccinations.

In the context of the study's findings, it became evident that a multifaceted approach is imperative to enhance children's immunization coverage in Afghanistan. The significance of these results underscored the urgency of addressing various aspects of the immunization landscape in the region. First and foremost, there is a compelling need for a targeted focus on policy implementation and outreach strategies, particularly in urban areas, to ensure that vaccination services are universally accessible. These efforts should be geared towards achieving equitable immunization coverage across diverse demographic groups, thereby mitigating disparities in vaccination rates [20]. Another critical dimension for future endeavors lies in the identification and rectification of misconceptions that influence vaccination decisions. The study underscored the importance of tailored education campaigns designed to counteract these misconceptions and enhance public understanding of the manifold benefits that immunization confers. Additionally, it is imperative to invest in the augmentation of healthcare infrastructure, with a particular emphasis on urban regions [21]. The expansion and fortification of clinics, vaccination centers, and outreach programs will be instrumental in facilitating improved access to vaccination services, ultimately contributing to higher coverage rates among the population [22].

Moreover, as we delve into prospects, there is a pressing need for research endeavors that probe deeper into the dynamics of vaccine hesitancy and the specific concerns held by parents and caretakers in Afghanistan. These studies will provide insights into the underlying causes of hesitancy, thereby serving as a foundation for tailored interventions to bolster confidence in immunization. Concurrently, a robust system of monitoring and evaluation must be established and maintained to continuously track the progress of vaccination programs. This ongoing assessment will be indispensable in identifying emerging challenges and adapting strategies to address them effectively [23].

Furthermore, an expanded scope of research is warranted to encompass maternal immunization coverage, with a particular focus on TT vaccinations. Inclusion of maternal immunization within the research agenda will contribute to a more comprehensive understanding of immunization efforts in the region. Thus, the multifaceted approach delineated here, incorporating policy implementation, misconception rectification, healthcare infrastructure enhancement, vaccine hesitancy research, monitoring and evaluation, and maternal immunization assessment, collectively constitutes a robust framework for bolstering children's immunization coverage in Afghanistan and enhancing the overall health outcomes of the population.

## 5. Conclusions

In conclusion, this study investigated the vaccination coverage among children aged 12–23 months in urban areas of 12 provinces and Kabul, Afghanistan. The findings revealed inadequate vaccination coverage, with only 37.29% of children being fully vaccinated, falling significantly short of the target of 80%. The study highlighted variations in coverage rates for different vaccines, with BCG achieving the highest coverage rate at 71%, while other vaccines like Penta-1, Penta-3, OPV, and Measles 1 had lower coverage rates ranging from 53% to 68%. The analysis at the provincial level demonstrated disparities in coverage rates between provinces, emphasizing the need for targeted interventions to address specific barriers and challenges. Factors influencing parents' decisions regarding vaccination included recognizing the importance of vaccination, the convenience of nearby healthcare facilities, effective outreach efforts, positive interactions with vaccinators, unawareness, lack of time, and loss of vaccination cards. It is crucial to address misconceptions and improve communication to promote vaccination. However, further studies are necessary, specifically focusing on hindering factors. The study's limitations include the absence of national and rural-level data and the lack of information on maternal immunization coverage. The study recommends setting realistic targets to achieve optimal vaccination coverage, implementing effective strategies, and conducting awareness campaigns to enhance vaccination coverage, decrease dropout rates, and address reasons for partial or no immunization.

## Figures and Tables

**Table 1:** Routine immunization implemented by the Ministry of Public Health, Afghanistan.

**Vaccines**	**Ages**					
	**As soon as possible after birth** ^a^	**6 weeks old**	**10 weeks old**	**14 weeks old**	**After 9 months**	**18 months**
BCG	X					
Hepatitis B	X					
OPV (0–4)	X	X	X	X	X	
Penta^b^ (1–3)		X	X	X		
PCV (1–3)		X	X	X		
Rotavirus (1, 2)		X	X			
IPV (1, 2)				X	X	
Measles 1					X	
Measles 2						X

^a^BCG (0–11 months, hepatitis B (birth dose) (after birth within 24 h), and OPV-0 (after births within 14 days)).

^b^Components of the Penta vaccine are diphtheria, pertussis, tetanus, Hep-B, and Hib.

**Table 2:** Sociodemographic factors associated with immunization among 12–23-month-old children in 12 provinces of Afghanistan, 2019.

**Main categories**	**Subcategories**	**Vaccinated # (%)**	**Unvaccinated # (%)**	**Overall # (%)**
Age group in months	12 to < 15	301 (32.19)	732 (30.02)	1033 (30.63)
	15 to < 18	234 (25.03)	546 (22.40)	780 (23.12)
	18 to < 21	213 (22.78)	633 (25.96)	846 (25.08)
	21 to < 24	187 (20.00)	527 (21.62)	714 (21.17)

Primary caretaker's sex	Male	476 (50.96)	1237 (50.74)	1713 (50.08)
	Female	458 (49.04)	1201 (49.26)	1659 (49.20)

Primary caretaker's literacy	Illiterate	554 (59.31)	1634 (67.10)	2188 (64.95)
	Literate	380 (40.69)	801 (32.90)	1181 (35.05)

Primary caretaker's education	Primary	101 (27.01)	201 (25.61)	302 (26.06)
	Secondary	84 (22.46)	190 (24.20)	274 (23.64)
	High school	95 (25.40)	203 (25.86)	298 (25.71)
	Higher education	69 (18.45)	120 (15.29)	189 (16.31)
	Others	25 (6.68)	71 (9.04)	96 (8.28)

Primary caretaker working outdoor	Yes	101 (10.87)	316 (13.11)	417 (12.49)
	No	828 (89.13)	2094 (86.89)	2922 (87.51)

Primary caretaker's relationship	Mother	785 (84.23)	1835 (75.64)	2622 (78.02)
	Father	60 (6.44)	227 (9.36)	289 (8.55)
	Grandmother	24 (2.58)	96 (3.96)	122 (3.57)
	Grandfather	5 (0.54)	33 (1.36)	40 (1.13)
	Others	58 (6.22)	235 (9.68)	295 (8.73)

**Table 3:** Immunization coverage by different antigens in urban settings, Afghanistan.

**SN**	**Vaccines**	**By card (** **n** **= 2421)**		**By history (** **n** **= 950)**		**Card + history** **(** **n** ** = 3382)**	
		**Number**	**%**	**Number**	**%**	**Number**	**%**
1	BCG	2375	70.22	25	0.74	2400	70.96
2	Hepatitis B	1740	65.22	109	4.09	1849	69.31
3	OPV-0	2063	61	211	6.24	2274	67.24
4	OPV-1	2302	68.07	20	0.59	2322	68.66
5	Penta-1	2292	67.77	8	0.24	2300	67.94
6	PCV1	2288	67.65	4	0.12	2292	67.8
7	Rota1	2171	64.19	28	0.83	2199	65.02
8	OPV-2	2141	63.31	27	0.8	2168	64.11
9	Penta-2	2140	63.28	7	0.21	2147	63.49
10	PCV2	2113	62.48	6	0.18	2119	62.66
11	Rota2	2041	60.35	31	0.92	2072	61.27
12	OPV-3	1999	59.11	29	0.86	2028	59.97
13	Penta-3	1995	58.99	3	0.09	1998	59.08
14	PCV3	1967	58.16	26	0.77	1993	58.93
15	OPV-4	1729	51.12	732	21.64	2461	72.76
16	Measles 1	1795	53.08	520	15.38	2315	68.46
17	IPV	1969	58.22	NA	NA	1969	58.22

## Data Availability

The dataset is available in ANPHI at MoPH and will be provided upon request and approval of ANPHI.

## Supporting Information

Supporting Information Additional supporting information can be found online in the Supporting Information section. Appendix 1 Questionnaire for EPI coverage survey. Appendix 2 Coverage of immunization by card, history, and differentiation of provinces.
