# Supplementary material for: Child Immunization Coverage in Urban Settings of Twelve Provinces Plus Kabul, Afghanistan, 2019
Source: Biomed Res Int. 2024 Aug 14;2024:5400013. doi: 10.1155/2024/5400013 (PMC11338657; doi:10.1155/2024/5400013)
Supplement: Supporting Information — Additional supporting information can be found online in the Supporting Information section. Appendix 1 Questionnaire for EPI coverage survey. Appendix 2 Coverage of immunization by card, history, and differentiation of provinces. [file 5400013.f1.docx]

**Appendix 1: Questionnaire for EPI coverage survey**

Islamic Republic of Afghanistan

Ministry of Public Health

Afghanistan National Public Health Institute

Questionnaire for EPI Coverage Survey

2019

*Name of province and its code: ----------------*

*Cluster number:*

*Name of village: --------------------------*

*Household number:*

*Name of household head and his/her mobile number: --------------------------------------------------*

*Date of interview: /-----/-----/--------------)*

*Time of the interview: /-----/-------/*

*Name of surveyor: Name of editor:*

*Signature of surveyor: Signature of editor:*

**Consent form**

I am …………… working in Ministry of Public Health .You are being asked to take part in a research study which may take 30 minutes. You are selected random in this survey and your participation in this study is voluntary and you can withdraw from the survey at any time. The purpose of this study is to find urban vaccination coverage and associated factors with coverage in {name city}. There is a questionnaire which includes demographic and immunization related question and you will be asked to answer the questions. Your participant to this study very important for obtain desirable results. There is no benefit or harm allocated for your participation in this study and your responses to this survey will be anonymous.

If you don’t want answer some questions tell I and I will go to next question .if you are willing to take part in this study.

May I start the interview?

1. I agree to participate in this survey

2. I can’t answer to all questions

3. Interviewer not present at home

4. I do not agree to participant in this survey

| **Demographic Questions** | | | |
| --- | --- | --- | --- |
| **#** | **Questions** | **Answers** | **Remarks** |
| 1 | What is your name (name of Parents) *optional | (Name) |  |
| 2 | Are you literate? | 1. Yes 2. No | If “No” go to question **4** |
| 3 | If yes, what is the highest level of school you attended? | 1. Primary (1 to sixth class of school) 2. Secondary (seventh to ninth) 3. High school (10^th^-12^th^) 4. Higher education 5. Madrasa 6. Other. _Please specify_ ------------- |  |
| 4 | Do you work outside? | 1. Yes 2. No |  |
| 5 | How many 12-23 months children do you have? | (Number) |  |
| 6 | What is the name of this child whose age is between 12-23 months? |  |  |
| 7 | Confirm the name of target child and ask how many months old is the child (Name)? | *date of birth: dd / mm / yyyy*  *……../……../…..*  *OR/and*  *Age in months: ________.............* |  |
| 8 | Record the sex of child | 1. Boy 2. Girl |  |

**Immunization questions:**

Copy vaccination date for each vaccine from card write ‘55 in ‘day' column if card shows that a vaccination was given, but no date is recorded. Write “66” if the month of the vaccination if not given.

| **Questions** | **Answers** | | **Remarks** | |
| --- | --- | --- | --- | --- |
| 10. Do you a have card (with yellow color) where (NAME)’S vaccinations are written down? | 1. Yes Seen 2. Yes Not seen 3. No | | If the answer is **“NO”** or **not seen the card**, go to question **“11”** | |
| BCG (Tuberculosis) | HEP 0 | | OPV0 | |
| /13_______/_______/______/ | /13_______/_______/______/ | | /13_______/_______/______/ | |
| PENTA1 | OPV1 | | PCV1 | |
| /13_______/_______/______/ | /13_______/_______/______/ | | /13_______/_______/______/ | |
| PENTA2 | OPV2 | | PCV2 | |
| /13_______/_______/______/ | /13_______/_______/______/ | | /13_______/_______/______/ | |
| ROTA 1 | PENTA3 | | OPV3 | |
| /13_______/_______/______/ | /13_______/_______/______/ | | /13_______/_______/______/ | |
| PCV3 | IPV | | Measles 1 | |
| /13_______/_______/______/ | /13_______/_______/______/ | | /13_______/_______/______/ | |
| OPV4 | ROTA2 | |  | |
| /13_______/_______/______/ | /13_______/_______/______/ | |  | |
| Q11: Please tell us that what motivated you in order to vaccinate you child? | 1. I understand the importance of vaccine 2. The health facility was close to our home 3. Outreach came to our village 4. Good behavior of vaccinator 5. Other. _Please specify_ ________________ | |  | |
| *Please review question above and check weather if all vaccines (BCG to Measles1) recorded.*  🞎 *Yes⇨ End the interview with this child*  🞎 *No ⇨ Ask questions bellow* | | | | |
| 12. Did [NAME] ever receive any vaccinations to prevent him/her from getting diseases, including vaccines received on national immunization day? | | 1. YES 2. No 3. Don’t know | | If the answer is “NO” go to question **30** |
| 12. Did [NAME] receive a BCG vaccination against tuberculosis that is an injection in the forearm that usually causes a scar? | | 1. Yes 2. No 3. Don’t know | | If the answer is “NO” go to question **15** |
| 13. if the answer is “Yes” or “Don’t know” please check the child for BCG scare at the left upper arm | | 1. Yes, BCG scare is present 2. No, BCG scare is not present | | If the answer is “NO” go to question **15** |
| 14. Where did [NAME] receive it? | | 1. Health Facility 2. Out-reach reach (vaccinators come to the centre of village/mosque) 3. Mobile Health team 4. Don’t know 5. Other _please Specify_----------- | |  |
| 15. Did (Name) receive HEP 0-dose that is given at birth (first 24 hours of life)? | | 1. Yes 2. No 3. Don’t know | |  |
| 16. Did [NAME] receive a polio vaccine, that is drops in the mouth after birth or 15 days after birth? | | 1. Yes 2. No 3. Don’t know | | If the answer is “NO” go to question **20** |
| 17. Where did [NAME] receive this first polio vaccine? | | 1. Health facility 2. Out-reach (vaccinators come to the centre of village/mosque) 3. Polio campaign (Vaccinator came to our door)..... 4. Mobile Health team 5. Don’t know 6. Other _Please specify_ ___________ | |  |
| 18. How many times was the polio vaccine received? | | Number of doses (Excluded birth dose) | |  |
| 19. Where did [NAME] receive these polio vaccines?  (Exclude birth dose, write number of doses for each location. Total number should be equal to number of dose in previous question ) | | 1. Health facility 2. Out-reach (vaccinators come to the centre of village/mosque) 3. Polio campaign (Vaccinator came to our door)..... 4. Mobile health team 5. Don’t know 6. Other _Please specify_ | |  |
| 20. Did [NAME] receive a Pentavalent vaccine that is an injection in the thigh usually given at the same time as the polio vaccine? | | 1. Yes 2. No 3. Don’t know (DK) | | If the answer is “NO” go to question **23** |
| 21. How many times was the Pentavalent vaccine given? | | \|___\|___\| | |  |
| 22. Where did [NAME] receive the last Pentavalent (vaccine? | | 1. Health Facility 2. Out-reach reach (vaccinators come to the centre of village/mosque) 3. Mobile Health team 4. Don’t know 5. Other _please Specify_----------- | |  |
| 23. Did [name] receive a MEASLES vaccine, that is a shot in the arm at the age of 9 months or older to prevent (him/her) from getting measles | | 1. Yes 2. No 3. Don’t know | | If the answer is “NO” or “Don’t know” go to question **25** |
| 24. How many times was the MEASLES vaccine given? | | \|___\|___\| | |  |
| 25. Did [NAME] receive a Vaccine against Pneumonia (PCV)? | | 1. Yes 2. No 3. Don’t know | | If the answer if “NO” or “Don’t know” go to question **27** |
| 26. How many times was the Pneumonia (PCV) vaccine given | | \|___\|___\| | |  |
| 27. Did [NAME] receive a vaccine drops in the mouth against diarrhea (ROTA)? | | 1. Yes 2. No 3. Don’t know | | If the answer if “NO” or “Don’t know” go to question **29** |
| 28. How many times was the Rota vaccine given. | | \|___\|___\| | |  |
| 29. Why you did not complete/continue vaccines of your children  **(for incomplete vaccines/Dropout)** | | 1. I wasn’t aware of the importance of second and third doses of vaccine 2. Vaccines side reaction after first dose 3. Long time gap between the first and second dose of the vaccine 4. Unavailability of vaccinator 5. No time for visiting HF 6. The Health facility was closed 7. The Health facility was far away from our home 8. Insecurity 9. Immigrated 10. The child was sick 11. The child was sick, although the child went to the HF but didn’t get the vaccine 12. Outreach service not visit the village 13. Vaccination card lost 14. HF did not advise for next doses during first visit 15. Other _please specify_ _____________ | | |
| 30. Ask the child’s mother or caretaker to give the most important reason why the child did not receive any immunizations in the series. Wait until the respondent answers in her own words **(Do not read the list of possible answers). (circle the reason (circle the related letter) closest to the answer given)** | | 1. Unaware of need for immunization 2. Place/time of immunization unknown 3. Fear of side reactions ‘ 4. Wrong ideas about contraindication 5. No faith in immunization 6. Rumors 7. Place of immunization too far 8. Insecurity 9. Immigrant issues 10. Vaccinator absent 11. Vaccine not available 12. Mother too busy 13. Family problem including illness of mother 14. Mother was not allow 15. Child ill-not brought 16. Child ill-brought out but not given immunization 17. Long waiting time 18. Other _please specify_ _________________________ | | |

**Appendix 2. Coverage of immunization by card, history, and differentiation of provinces**

| **Vaccine** | **Categories** | **Kabul** | | **Nangarhar** | | **Kandahar** | | **Farah** | | **Ghazni** | | **Helmand** | | **Khost** | | **Kunar** | | **Kunduz** | | **Nooristan** | | **Paktika** | | **Sarepul** | | **Zabul** | | **Total** | |
| --- | --- | --- | --- | --- | --- | --- | --- | --- | --- | --- | --- | --- | --- | --- | --- | --- | --- | --- | --- | --- | --- | --- | --- | --- | --- | --- | --- | --- | --- |
|  |  | **#** | **%** | **#** | **%** | **#** | **%** | **#** | **%** | **#** | **%** | **#** | **%** | **#** | **%** | **#** | **%** | **#** | **%** | **#** | **%** | **#** | **%** | **#** | **%** | **#** | **%** | **#** | **%** |
| **BCG** | By Card | 498 | 69.75 | 199 | 98.03 | 313 | 70.02 | 118 | 69.41 | 177 | 81.57 | 163 | 67.63 | 144 | 66.67 | 88 | 40.00 | 160 | 77.29 | 78 | 82.11 | 96 | 42.86 | 197 | 93.81 | 144 | 66.06 | 2375 | 70.22 |
|  | By History | 2 | 0.28 | 0 | 0.00 | 5 | 1.12 | 3 | 1.76 | 1 | 0.46 | 1 | 0.41 | 2 | 0.93 | 4 | 1.82 | 3 | 1.45 | 0 | 0.00 | 2 | 0.89 | 2 | 0.95 | 0 | 0.00 | 25 | 0.74 |
|  | Card+History | 500 | 70.03 | 199 | 98.03 | 318 | 71.14 | 121 | 71.18 | 178 | 82.03 | 164 | 68.05 | 146 | 67.59 | 92 | 41.82 | 163 | 78.74 | 78 | 82.11 | 98 | 43.75 | 199 | 94.76 | 144 | 66.06 | 2400 | 70.96 |
|  | Not vaccinated | 214 | 30 | 4 | 1.97 | 129 | 28.86 | 49 | 28.82 | 39 | 17.97 | 77 | 31.95 | 70 | 32.41 | 128 | 58.18 | 44 | 21.26 | 17 | 17.89 | 126 | 56.25 | 11 | 5.24 | 74 | 33.94 | 982 | 29.04 |
| **Rota2** | By Card | 459 | 64.29 | 182 | 89.66 | 254 | 56.82 | 101 | 59.41 | 168 | 77.42 | 122 | 50.62 | 95 | 43.98 | 87 | 39.55 | 126 | 60.87 | 47 | 49.47 | 88 | 39.29 | 181 | 86.19 | 131 | 60.09 | 2041 | 60.35 |
|  | By History | 7 | 0.98 | 0 | 0.00 | 3 | 0.67 | 3 | 1.76 | 1 | 0.46 | 5 | 2.07 | 1 | 0.46 | 1 | 0.45 | 1 | 0.48 | 6 | 6.32 | 3 | 1.34 | 0 | 0.00 | 0 | 0.00 | 31 | 0.92 |
|  | Card+History | 466 | 65.27 | 182 | 89.66 | 257 | 57.49 | 104 | 61.18 | 169 | 77.88 | 127 | 52.70 | 96 | 44.44 | 88 | 40.00 | 127 | 61.35 | 53 | 55.79 | 91 | 40.63 | 181 | 86.19 | 131 | 60.09 | 2072 | 61.27 |
|  | Not vaccinated | 248 | 34.73 | 21 | 10.34 | 190 | 42.51 | 66 | 38.82 | 48 | 22.12 | 114 | 47.30 | 120 | 55.56 | 132 | 60.00 | 80 | 38.65 | 42 | 44.21 | 133 | 59.38 | 29 | 13.81 | 87 | 39.91 | 1310 | 38.73 |
| **OPV3** | By Card | 475 | 66.53 | 181 | 89.16 | 256 | 57.27 | 96 | 56.47 | 154 | 70.97 | 121 | 50.21 | 70 | 32.41 | 86 | 39.09 | 113 | 54.59 | 57 | 60.00 | 89 | 39.73 | 173 | 82.38 | 128 | 58.72 | 1999 | 59.11 |
|  | By History | 3 | 0.42 | 0 | 0.00 | 6 | 1.34 | 2 | 1.18 | 2 | 0.92 | 3 | 1.24 | 2 | 0.93 | 1 | 0.45 | 3 | 1.45 | 0 | 0.00 | 1 | 0.45 | 4 | 1.90 | 2 | 0.92 | 29 | 0.86 |
|  | Card+History | 478 | 66.95 | 181 | 89.16 | 262 | 58.61 | 98 | 57.65 | 156 | 71.89 | 124 | 51.45 | 72 | 33.33 | 87 | 39.55 | 116 | 56.04 | 57 | 60.00 | 90 | 40.18 | 177 | 84.29 | 130 | 59.63 | 2028 | 59.96 |
|  | Not vaccinated | 236 | 33.05 | 22 | 10.84 | 185 | 41.39 | 72 | 42.35 | 61 | 28.11 | 117 | 48.55 | 144 | 66.67 | 133 | 60.45 | 91 | 43.96 | 38 | 40.00 | 134 | 59.82 | 33 | 15.71 | 88 | 40.37 | 1354 | 40.04 |
| **Penta3** | By Card | 470 | 65.83 | 178 | 87.68 | 259 | 57.94 | 97 | 57.06 | 156 | 71.89 | 118 | 48.96 | 70 | 32.41 | 86 | 39.09 | 112 | 54.11 | 57 | 60.00 | 90 | 40.18 | 173 | 82.38 | 129 | 59.17 | 1995 | 58.99 |
|  | By History | 0 | 0.00 | 0 | 0.00 | 0 | 0.00 | 0 | 0.00 | 0 | 0.00 | 0 | 0.00 | 1 | 0.46 | 1 | 0.45 | 0 | 0.00 | 0 | 0.00 | 0 | 0.00 | 1 | 0.48 | 0 | 0.00 | 3 | 0.09 |
|  | Card+History | 470 | 65.83 | 178 | 87.68 | 259 | 57.94 | 97 | 57.06 | 156 | 71.89 | 118 | 48.96 | 71 | 32.87 | 87 | 39.55 | 112 | 54.11 | 57 | 60.00 | 90 | 40.18 | 174 | 82.86 | 129 | 59.17 | 1998 | 59.08 |
|  | Not vaccinated | 244 | 34.17 | 25 | 12.32 | 188 | 42.06 | 73 | 42.94 | 61 | 28.11 | 123 | 51.04 | 145 | 67.13 | 133 | 60.45 | 95 | 45.89 | 38 | 40.00 | 134 | 59.82 | 36 | 17.14 | 89 | 40.83 | 1384 | 40.92 |
| **IPV3** | By Card | 478 | 66.95 | 120 | 59.11 | 242 | 54.14 | 104 | 61.18 | 80 | 36.87 | 127 | 52.70 | 130 | 60.19 | 150 | 68.18 | 110 | 53.14 | 33 | 34.74 | 131 | 58.48 | 124 | 59.05 | 140 | 64.22 | 1969 | 58.22 |
|  | By History | 0 | 0.00 | 0 | 0.00 | 0 | 0.00 | 0 | 0.00 | 0 | 0.00 | 0 | 0.00 | 0 | 0.00 | 0 | 0.00 | 0 | 0.00 | 0 | 0.00 | 0 | 0.00 | 0 | 0.00 | 0 | 0.00 | 0 | 0.00 |
|  | Card+History | 478 | 66.95 | 120 | 59.11 | 242 | 54.14 | 104 | 61.18 | 80 | 36.87 | 127 | 52.70 | 130 | 60.19 | 150 | 68.18 | 110 | 53.14 | 33 | 34.74 | 131 | 58.48 | 124 | 59.05 | 140 | 64.22 | 1969 | 58.22 |
|  | Not vaccinated | 236 | 33.05 | 83 | 40.89 | 205 | 45.86 | 66 | 38.82 | 137 | 63.13 | 114 | 47.30 | 86 | 39.81 | 70 | 31.82 | 97 | 46.86 | 62 | 65.26 | 93 | 41.52 | 86 | 40.95 | 78 | 35.78 | 1413 | 41.78 |
| **Measles1** | By Card | 437 | 61.20 | 111 | 54.68 | 225 | 50.34 | 98 | 57.65 | 67 | 30.88 | 116 | 48.13 | 115 | 53.24 | 144 | 65.45 | 91 | 43.96 | 31 | 32.63 | 120 | 53.57 | 120 | 57.14 | 120 | 55.05 | 1795 | 53.08 |
|  | By History | 122 | 17.09 | 48 | 23.65 | 67 | 14.99 | 25 | 14.71 | 47 | 21.66 | 32 | 13.28 | 17 | 7.87 | 32 | 14.55 | 28 | 13.53 | 13 | 13.68 | 19 | 8.48 | 32 | 15.24 | 38 | 17.43 | 520 | 15.38 |
|  | Card+History | 559 | 78.29 | 159 | 78.33 | 292 | 65.32 | 123 | 72.35 | 114 | 52.53 | 148 | 61.41 | 132 | 61.11 | 176 | 80.00 | 119 | 57.49 | 44 | 46.32 | 139 | 62.05 | 152 | 72.38 | 158 | 72.48 | 2315 | 68.46 |
|  | Not vaccinated | 155 | 21.71 | 44 | 21.67 | 155 | 34.68 | 47 | 27.65 | 103 | 47.47 | 93 | 38.59 | 84 | 38.89 | 44 | 20.00 | 88 | 42.51 | 51 | 53.68 | 85 | 37.95 | 58 | 27.62 | 60 | 27.52 | 1067 | 31.55 |
